# Catastrophic health expenditures, insurance churn, and non-employment among women with breast cancer

**DOI:** 10.1093/jncics/pkae006

**Published:** 2024-02-08

**Authors:** Nicholas L Berlin, Benjamin B Albright, Haley A Moss, Anaeze C Offodile

**Affiliations:** Section of Plastic Surgery, Department of Surgery, University of Michigan, Ann Arbor, MI, USA; Division of Gynecologic Oncology, Department of Obstetrics and Gynecology, University of North Carolina, Chapel Hill, NC, USA; Division of Gynecologic Oncology, Duke University School of Medicine, Durham, NC, USA; Plastic and Reconstructive Surgery Service, Department of Surgery, Memorial Sloan Kettering Cancer Center, New York, NY, USA

## Abstract

**Background:**

Breast cancer treatment and survivorship entails a complex and expensive continuum of subspecialty care. Our objectives were to assess catastrophic health expenditures, insurance churn, and non-employment among women younger than 65 years who reported a diagnosis of breast cancer. We also evaluated changes in these outcomes related to implementation of the Affordable Care Act.

**Methods:**

The data source for this study was the Medical Expenditure Panel Survey (2005-2019), which is a national annual cross-sectional survey of families, providers, and insurers in the United States. To assess the impact of breast cancer, comparisons were made with a matched cohort of women without cancer. We estimated predicted marginal probabilities to quantify the effects of covariates in models for catastrophic health expenditures, insurance churn, and non-employment.

**Results:**

We identified 1490 respondents younger than 65 years who received care related to breast cancer during the study period, representing a weight-adjusted annual mean of 1 062 129 patients. Approximately 31.8% of women with breast cancer reported health expenditures in excess of 10% of their annual income. In models, the proportion of women with breast cancer who experienced catastrophic health expenditures and non-employment was inversely related to increasing income. During Affordable Care Act implementation, mean number of months of uninsurance decreased and expenditures increased among breast cancer patients.

**Conclusions:**

Our study underscores the impact of breast cancer on financial security and opportunities for patients and their families. A multilevel understanding of these issues is needed to design effective and equitable strategies to improve quality of life and survivorship.

Breast cancer (BC) diagnosis and treatment encompasses a complex continuum of subspecialty care across multiple treatment settings. This often comes at a time when women are of working age, accumulating savings, and likely to have dependents. Nearly half of women with BC experience at least moderate financial distress, which adversely affects treatment choices, quality of life, patient satisfaction, medication adherence, bankruptcy rates, and even mortality (1-9). Termed “financial toxicity” (FT), this multifactorial experience among BC survivors has been the focus of intense research and policy interest with respect to creating theoretical frameworks and mitigation strategies (6,10).

In this context, the financial and workforce implications of undergoing BC treatment are of increasing concern for patients, health-care providers, and policymakers (1,11,12). Despite increasing awareness of the adverse sequelae of FT, there is considerable need to deepen our understanding of the prevalence and inter-relationship of catastrophic health expenditures (CHE), health insurance changes, and adverse employment outcomes among the BC subpopulation (13). This is because BC patients, relative to other solid tumor patients, have a lower median age at diagnosis, are more likely to experience treatment-related work interruption, have experienced vastly improved overall survival outcomes due to early detection and advances in treatment approaches, and are at elevated risk for second primary cancers (14-16). These characteristics serve to uniquely intensify the vulnerability, presentation, and duration of FT among women with BC younger than 65 years in the United States.

Several provisions of the Affordable Care Act (ACA), including Medicaid expansions, elimination or reduced cost sharing for evidence-based preventive cancer screening, creation of a health-care marketplace, and coverage of clinical trial participation have been shown to materially improve cancer care access, insurance protections, and clinical outcomes in BC patients (17-19). Yet gaps in coverage persist, and patients continue to grapple with high out-of-pocket (OOP) spending, work productivity and job loss, and consequent financial toxicities related to BC care receipt.

For the present study, our primary objectives were to assess prevalence and determinants of CHE, insurance churn, and non-employment among women younger than 65 years with medical expenditures associated with a BC diagnosis in a given year. We also evaluated changes in insurance coverage and CHE related to ACA implementation.

## Methods

### Study data

Our primary data source was the Medical Expenditure Panel Survey (MEPS) data from the Agency of Healthcare Research and Quality. MEPS is an annual cross-sectional panel survey of families, medical providers, and employers including approximately 13 000 families comprising 30 000 individuals each year (20,21). These data are a weighted sample that can be used to estimate population-level statistics of medical expenditures, monthly insurance coverage, employment status, and medical care usage.

The dataset reflects survey data from 2005 to 2019, which spanned the years before (2005-2009), during (2010-2013), and after (2014-2019) implementation of the ACA. Our study time horizon was limited to 2019 due to data availability and an attempt to mitigate unmeasured confounding brought about by the significant workforce and labor participation disruptions of the COVID-19 pandemic. We excluded respondents aged 65 years and older due to the near-universal coverage with Medicare and structural differences with the other payer categories (ie, no OOP maximum, significant heterogeneity in use of drug coverage via supplemental insurance, and growth of Medicare Advantage enrollment). We also excluded respondents less than 18 years because of a lower likelihood to seek employment and reduced risk for both BC and our outcomes of interest. The study was limited to women with BC who had medical expenditures associated with a BC diagnosis in a given year. To facilitate this, we used corresponding International Classification of Disease (ICD) code (International Classification of Diseases, Ninth Revision [ICD-9], Clinical Classifications Software codes 24; International Classification of Diseases, Tenth Revision [ICD- 10], codes C50x).

### Independent and dependent variables

Independent variables for our multivariable analyses included the following: age, race (White, Black, other, or mixed), Hispanic ethnicity, low educational attainment (no high school degree or equivalent), marital status, family size (total count of people), presence of self-reported comorbid conditions, and family income in relation to the federal poverty level during each individual year of the survey (federal poverty level [FPL]: ≤138%, 139%-250%, 251%-400%, >400%). The race category of “other” consisted of any non-White and non-Black races, specifically assessed as American Indian or Alaska Native, Asian, Native Hawaiian or Pacific Islander, and multiple races. Comorbidities are collected by MEPS through self-report of ever being diagnosed any of the following conditions: hypertension, hypercholesterolemia, coronary artery disease, angina, stroke, myocardial infarction, other heart disease, diabetes, emphysema, asthma, and arthritis.

We report three primary outcomes of interest: (1) CHE (2) insurance churn, and (3) non-employment.

For CHE, we based our assessment on those defined by the Commonwealth Fund (22). As health expenses are typically shared among a family and MEPS allows for family-level analysis, we assessed for CHE by the ratio of family annual OOP health spending to family income. Health expenses exceeding 10% of income, excluding premiums, are classified as financially catastrophic (or 5% of income for the poor) (22). We considered expenses with and without premiums and assessed according to the 5% and 10% thresholds. Lastly, the burden of annual health expenditures was also considered in terms of family spending and family incomes, consistent with published studies (23,24).

Insurance churn variables were defined as the loss of insurance coverage (moving from any insurance to uninsurance), change in coverage, including change in insurance type (eg, private coverage to Medicaid coverage), any uninsurance (at least 1 month), and total annual months of uninsurance (range, 0-12 months), also consistent with previous MEPS studies (24,25).

For non-employment outcomes, respondents were asked about current employment status and whether they have a job or a job to return to three times annually. Respondents who identified as retired or having never worked were excluded from analysis of employment outcomes. We also assessed for annual risks of at least part-year non-employment, full-year non-employment, job change or loss, and missed days of work or non-employment reported as secondary to illness or injury.

### Statistical analysis

To assess the impact of BC on outcomes, we created a matched cohort from survey respondents, over the same time horizon, without a cancer diagnosis. We conducted 2:1 nearest-neighbor propensity matching without replacement and with maximum caliper of 0.001. Patients were matched on survey year, survey weight, and demographic characteristics (age, female sex, race, ethnicity, educational attainment, marital status, family size, comorbidity burden count, and family income category).

All analyses were performed using survey weighting to estimate outcomes for the broader US population, with standard errors accounting for complex survey design and variance estimation using Taylor series linearization. Since patients in the primary analytic cohort had a cancer diagnosis, presence of a cancer diagnosis was excluded from the total count of comorbidities. We used the adjusted Wald test to compare outcomes between subgroups and over time for statistical significance. Data were pooled across years to increase sample size and improve the precision of point estimates. We also estimated predicted marginal probabilities to quantify the effects of covariates in multivariable models. For multivariable models, comorbidities were dichotomized (0 vs 1 vs ≥2). We considered two-tailed *P* values less than .05 to be statistically significant. The Strengthening the Reporting of Observational Studies in Epidemiology (STROBE) guidelines for observational research were followed (26). Analyses were conducted with Stata statistical software (version 15.1; StataCorp, College Station, TX). These data are deidentified and publicly available; therefore, the study was deemed exempt by the University of Michigan Institutional Review Board.

## Results

### Sample characteristics

We identified a total of 1490 MEPS respondents younger than 65 years reporting care associated with a BC diagnosis in a given year from 2005 to 2019, which extrapolates to an estimated annual mean of 1 062 129 female patients (95% Confidence Interval [CI] = 957 573 to 1 166 686; mean age, 53.8 years [95% CI = 53.1 to 54.6 years]) in the United States with health-care use associated with a BC diagnosis in the given year. The propensity-score-matched comparison cohort of female patients without cancer included 2980 respondents and was balanced on all matching characteristics (Table 1).

**Table 1.** Demographic characteristics for breast cancer patients younger than 65 years in the United States, relative no cancer population, Medical Expenditure Panel Survey, 2005-2019

	**Breast cancer** (n = 1490) weighted mean (95% CI)	**No cancer** (n = 2980) weighted mean (95% CI)	*P*
**Annual population**	1 062 129 (957 573 to 1 166 686)	2 094 244 (1 971 571 to 2 216 917)	
**Age** (years)	53.8 (53.1 to 54.6)	53.6 (53.1 to 54.0)	.506
**Female sex** (%)	100%	100%	
**Race** (%)			
* Black*	12.8% (10.2 to 15.3)	12.3% (10.7 to 14.0)	.750
* Other/mixed* ^a^	6.5% (4.7 to 8.4)	7.2% (5.8 to 8.6)	.524
* White*	80.7% (77.6 to 83.7)	80.4% (78.3 to 82.6)	.903
**Hispanic ethnicity** (%)	9.4% (6.9 to 11.9)	9.3% (7.7 to 10.9)	.944
**Low education** ^b^ (%)	7.0% (4.8 to 9.2)	5.5% (4.6 to 6.3)	.186
**Married** (%)	65.3% (60.9 to 69.7)	63.4% (60.8 to 66.0)	486
**Family size** (n)	2.4 (2.3 to 2.5)	2.4 (2.4 to 2.5)	.982
**Family income** (%)			
* ≤138% FPL*	12.5% (9.9 to 15.1)	12.5% (11.0 to 14.1)	.996
* 139%-249% FPL*	13.2% (11.1 to 15.4)	14.7% (13.0 to 16.4)	.286
* 250%-400% FPL*	19.5% (16.2 to 22.7)	17.5% (15.6 to 19.5)	.358
* >400% FPL*	54.8% (50.5 to 59.2)	55.2% (52.6 to 57.8)	.870
**Comorbid diagnoses** ^c^ (n)	1.4 (1.3 to 1.5)	1.3 (1.2 to 1.4)	.606

a Other includes any non-White and non-Black races, specifically assessed as American Indian or Alaska Native, Asian, Native Hawaiian or Pacific Islander, and multiple races. CI = confidence interval; FPL = federal poverty level.

b Low education indicates no post-high school degree or equivalent.

c Count of self-reported noncancer comorbid conditions among hypertension, hypercholesterolemia, coronary artery disease, angina, stroke, myocardial infarction, other heart disease, diabetes, emphysema, asthma, and arthritis.

### Catastrophic health expenditures

Compared to women without cancer, women with BC were more likely to report expenditures (including premiums) that were in excess of 10% of annual household income (31.8% vs 25.9%, *P* = .008) (Table 2). More than half (50.4%) of women with annual household incomes less than 138% FPL reported expenditures (including premiums) that were in excess of 10% of annual income (Table 3). In contrast, for women with annual household income of at least 400% FPL, only 21.2% experienced expenditures in excess of 10% of annual household income (Table 3). In multivariable models, the proportion of women with BC who experienced CHE was inversely related to annual income (Table 4). For example, the predicted probability of CHE decreased as income increased (52.1%, 46.4%, 33.8%, and 17.6% for household incomes of ≤138% FPL, 139-249% FPL, 250-400% FPL, and >400% FPL) (*P* < .001).

**Table 2.** Unadjusted rates of catastrophic health expenditures, insurance churn, and non-employment among breast cancer patients younger than 65 years in the United States, overall comparison to patients without cancer, Medical Expenditure Panel Survey, 2005-2019

Outcome	**Breast cancer** (n = 1490) weighted mean (95% CI)	**No cancer** (n = 2980) weighted mean (95% CI)	*P*
**Catastrophic health expenditures** ^a^			
* * **Excluding premiums**			
* >10% income*	17.4% (14.8 to 20.1)	13.3% (11.7 to 14.9)	.008
* >5% income*	30.7% (27.4 to 34.1)	22.3% (20.2 to 24.3)	<.001
* * **Including premiums**			
* >10% income*	31.8% (28.1 to 35.4)	25.9% (23.7 to 28.2)	.008
* >5% income*	57.1% (53.1 to 61.1)	47.7% (45.2 to 50.1)	<.001
**Insurance churn**			
* Insurance loss*	4.9% (3.0 to 6.8)	5.7% (4.3 to 7.0)	.492
* Insurance change*	14.1% (11.4 to 16.7)	15.6% (13.8 to 17.4)	.306
* Any uninsurance*	11.3% (8.5 to 14.1)	18.0% (16.2 to 19.9)	<.001
* Uninsured all year*	3.4% (2.2 to 4.7)	9.6% (8.2 to 10.9)	<.001
**Non-employment**			
* Full-year non-employment*	21.4% (17.3 to 25.5)	17.2% (15.1 to 19.3)	.058
* Part-year non-employment*	30.5% (26.0 to 35.1)	27.1% (24.7 to 29.6)	.169
* Illness-related non-employment*	14.5% (11.1 to 18.0)	9.6% (7.9 to 11.2)	.009
* Job change/loss*	11.4% (8.8 to 14.0)	11.6% (9.9 to 13.5)	.871
* Missed workdays (n)*	5.7 (4.8 to 6.6)	2.7 (2.3 to 3.1)	<.001

a Calculated as ratio of family out-of-pocket expenditures (including or excluding premiums as indicated) divided by family income. CI = confidence interval.

**Table 3.** Unadjusted rates of catastrophic health expenditures, insurance churn, and non-employment among breast cancer patients younger than 65 years in the United States, by patient-reported income relative to federal poverty level, Medical Expenditure Panel Survey, 2005-2019

Outcome	≤138% FPL	139%-249% FPL	250%-399% FPL	≥400% FPL
Sample n	277	265	295	653
Annual estimated N	133 004	140 495	206 261	582 370
**Catastrophic health expenditures** ^a^				
**Excluding premiums**				
*>10% income (%)*	39.5%	26.4%*	16.4%	10.6%
*>5% income (%)*	49.2%	47.5%*	36.5%*	20.5%*
**Including premiums**				
*>10% income (%)*	50.4%	49.9%*	37.1%	21.2%*
*>5% income (%)*	59.1%	70.7%	71.0%*	48.4%*
**Insurance churn**				
*Insurance loss (%)*	4.9%	4.6%	7.1%	4.2%
*Insurance change (%)*	20.9%	18.9%	17.8%	10.0%
*Any uninsurance (%)*	16.1%**	13.5%**	17.5%	7.4%
*Uninsured all year (%)*	4.5%**	5.4%**	6.5%	1.6%**
**Non-employment**				
*Full-year non-employment (%)*	56.2%	28.2%	20.9%*	13.3%
*Part-year non-employment (%)*	70.2%	42.0%	29.9%	20.6%
*Illness-related non-employment (%)*	54.8%*	23.2%	13.8%*	5.1%
*Job change/loss (%)*	15.8%	14.6%	11.0%	10.0%
*Missed workdays (n)*	3.7	4.6*	7.7*	5.7*

a Ratio of annual family level out-of-pocket spending toward health-care expenses +/− insurance premiums to family income. FPL = federal poverty level.

* *P* < .05, higher risk for breast cancer patients vs noncancer patients.

** *P* < .05, lower risk for breast cancer patients vs noncancer patients.

**Table 4.** Predicted probabilities of catastrophic health expenditures, insurance churn, and non-employment among breast cancer patients younger than 65 years in the United States, Medical Expenditure Panel Survey, 2005-2019

	Model #1	Model #2	Model #3
	Catastrophic health expenditures (>10% of income, including premiums) % (95% CI)	Any insurance loss % (95% CI)	Part year non-employment % (95% CI)
**Age**			
18-35 years	28.4% (16.8 to 40.0)	8.7% (0.0 to 17.3)	33.4% (21.3 to 45.4)
36-50 years	26.5% (22.1 to 30.9)	4.9% (2.6 to 7.4)	32.1% (27.6 to 36.6)
50-64 years	33.2% (30.2 to 36.1)	5.0% (3.5 to 6.6)	37.2% (33.9 to 40.5)
**Race**			
* White*	34.3% (31.5 to 37.2)	5.4% (3.9 to 6.8)	35.0% (32.0 to 38.0)
* Black*	21.6% (17.2 to 26.0)	4.3% (1.7 to 6.9)	36.8% (31.1 to 42.7)
*Other*	31.6% (23.6 to 39.7)	–	34.9% (26.2 to 43.5)
**Hispanic ethnicity**			
No	33.7% (31.0 to 36.3)	5.1% (3.7 to 6.5)	35.5% (32.8 to 38.3)
Yes	20.4% (15.5 to 25.2)	5.3% (2.1 to 8.5)	34.9% (28.4 to 41.3)
**Education**			
No high school	21.1% (15.0 to 27.2)	3.1% (0.0 to 6.4)	41.8% (32.8 to 50.9)
High school degree	30.4% (26.9 to 33.9)	5.9% (3.8 to 8.0)	34.5% (30.8 to 38.3)
College degree	34.3% (30.7 to 37.8)	4.9% (3.1 to 6.7)	35.0% (31.2 to 38.7)
**Married**			
No	28.4% (24.4 to 32.3)	4.1% (2.3 to 6.0)	28.5% (24.6 to 32.4)
Yes	33.2% (29.7 to 36.7)	6.1% (3.8 to 8.4)	40.7% (37.0 to 44.4)
**Family size**			
1	28.2% (22.1 to 34.2)	7.4% (2.7 to 12.1)	37.4% (30.1 to 44.8)
2	37.3% (33.4 to 41.2)	5.3% (3.2 to 7.4)	35.6% (31.6 to 39.6)
≥3	26.3% (22.5 to 30.0)	4.2% (2.3 to 6.0)	34.5% (30.6 to 38.4)
**Family income**			
* ≤138% FPL*	52.1% (45.6 to 58.6)	6.7% (2.7 to 10.8)	76.8% (70.5 to 83.0)
* 139%-249% FPL*	46.4% (40.4 to 52.5)	6.0% (2.7 to 9.3)	46.9% (40.0 to 53.8)
* 250%-400% FPL*	33.8% (28.5 to 39.1)	7.0% (3.8 to 10.3)	29.0% (23.4 to 34.7)
* >400% FPL*	17.6% (14.8 to 20.4)	3.6% (2.1 to 5.2)	19.6% (16.2 to 23.0)
**Comorbid diagnoses** ^a^			
0	29.7% (25.4 to 33.9)	6.7% (4.0 to 9.4)	30.3% (26.1 to 34.5)
1	34.3% (29.9 to 38.8)	5.9% (3.4 to 8.5)	33.1% (28.5 to 37.8)
≥2	30.0% (26.3 to 33.7)	3.5% (1.9 to 5.1)	41.9% (37.5 to 46.4)

a Count of self-reported noncancer comorbid conditions among hypertension, hypercholesterolemia, coronary artery disease, angina, stroke, myocardial infarction, other heart disease, diabetes, emphysema, asthma, and arthritis. CI = confidence interval; FPL = federal poverty level.

### Insurance churn

In comparison to women without cancer, those with BC had lower rates of any uninsurance (11.3% [95% CI = 8.5% to 14.1%] vs 18.0% [95% CI = 16.2% to 19.9%] per year; *P* = .001) and uninsurance all year (3.4% [95% CI = 2.2% to 4.7%] vs 9.6% [95% CI = 8.2% to 10.9%] per year; *P* < .001). Approximately 17.5% of women with BC and annual incomes 250% to 399% FPL spent at least 1 month uninsured in any given year (Table 3). Annual household income was not associated with any insurance loss among BC patients in multivariable models (Table 4).

### Employment outcomes

Approximately 30.5% (95% CI = 26.0% to 35.1%) of women with BC experienced at least part-year non-employment (Table 2). In comparison to women without cancer, women with BC were more likely to experience illness related non-employment (14.5% vs 9.6%, *P* = .009), and they reported more missed workdays (5.7 days vs 2.7 days) (*P* < .001) (Table 2). In our subgroup analysis across income levels, the proportion of women who reported full-year non-employment decreased from 56.2% for women with annual incomes less than 138% FPL to 13.3% for women with annual incomes more than 400% FPL (Table 3). This inverse association between income and part-year non-employment remained significant in multivariable models (Table 4).

### Trends over time

**Figure 1.**
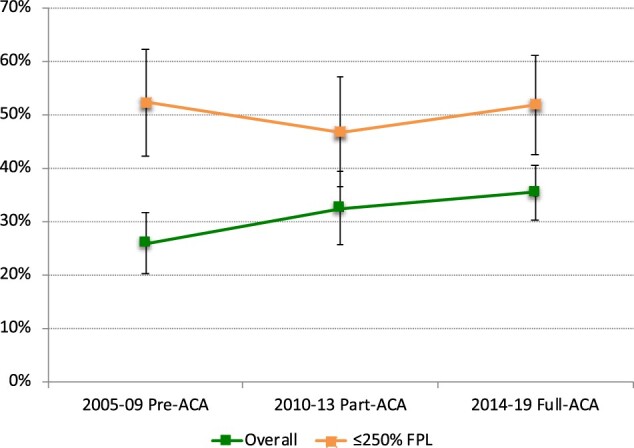
Time trends in catastrophic health expenditures (including premiums) relative to Affordable Care Act implementation among women younger than 65 years with breast cancer younger than 65 years, 2005-2019. ACA = Affordable Care Act; FPL = federal poverty level. Approximately 25.9% experienced health expenditures (including premiums) that were in excess of 10% of their annual income in 2005-2009, 32.5% in 2010-2013, and 35.4% in 2013-2019 (*P* = .015 for comparison of 2013-2019 to 2005-2009).

**Figure 2.**
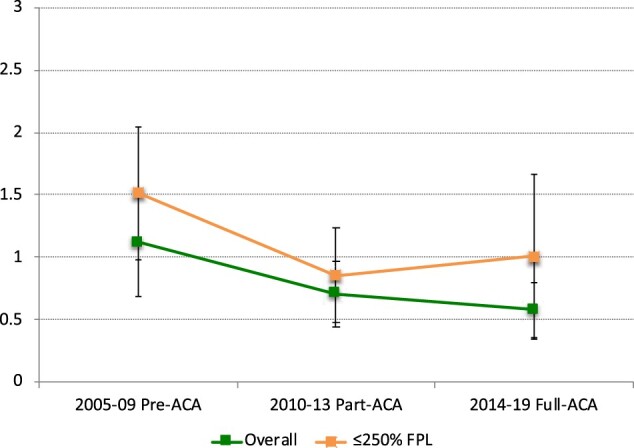
Time trends in insurance relative to Affordable Care Act implementation among women younger than 65 years with breast cancer, 2005-2019. ACA = Affordable Care Act; FPL = federal poverty level. On average, women with BC reported 1.1 months of uninsurance in 2005-2009, 0.71 months of uninsurance in 2010-2013, and 0.60 months of uninsurance in 2013-2019 (*P* < .031 for comparison of 2013-2019 to 2005-2009).

In our analysis of trends over time, there was a decrease in the mean annual total months of uninsurance among women with BC (Figure 1). On average, women with BC reported 1.1 months of uninsurance in 2005-2009, 0.71 months of uninsurance in 2010-2013, and 0.60 months of uninsurance in 2013-2019 (*P* < .031 for comparison of 2013-2019 to 2005-2009). However, the proportion of patients who reported health expenditures (including premiums) that were in excess of 10% of annual income increased over time. For example, approximately 25.9% experienced expenditures (including premiums) that were in excess of 10% of their annual income in 2005-2009, 32.5% in 2010-2013, and 35.4% in 2013-2019 (*P* = .015 for comparison of 2013-2019 to 2005-2009) (Figure 2).

## Discussion

Among women with incident BC in the United States, treatment-related hardship is a significant problem (1,11-13,27-30). In the present study, we demonstrated three important findings related to CHE, insurance churn, and non-employment. First, relative to similar women without cancer, women with BC are more likely to experience CHE and non-employment while also having lower rates of uninsurance. Second, higher annual household income was associated with a lower-likelihood CHE and non-employment among BC patients. Finally, we demonstrate that although months of uninsurance among women with BC decreased over time as they relate to ACA implementation, CHE have continued to increase. Taken together, these findings suggest that women with BC, and specifically those with lower household income, are at an increased risk for adverse financial and employment outcomes; suggesting that the policy provisions set in place by the Affordable Care Act are not sufficiently protective.

Previous investigators have shown how cancer patients typically face higher OOP expenses than those with other chronic conditions (23, 31). BC patients likely have lower rates of uninsurance than similar patients without cancer because cancer patients need insurance coverage to pay for care and are incentivized to pursue it. However, in recent years there has been a proliferation of high-deductible health plans (HDHPs) (32,33). We estimate that the rate of underinsurance over the study period was 17.4% (Table 2), which was statistically higher than for matched controls without cancer (13.3%) (*P* < .001) (34). Additionally, for BC patients with annual household incomes less than 138%, we reported the highest rates of underinsurance (49.2%) (Table 3). This is salient because low-income BC patients with HDHPs experienced significantly more suboptimal outcomes, eg, delays in time to breast imaging, first biopsy, and diagnosis of incident cancer (35).

That CHE and employment outcomes are affected by annual household income is consistent with previous studies on FT among BC patients (2,27,36,37). Other studies have even demonstrated an association between income and survival after treatment for cancer (38). For BC patients whose household income is not strictly dependent on their own employment (ie, spousal or caregiver support), the impact of OOP costs and lost wages from non-employment is likely to be lower. These associations reflect the disproportionate financial burden of BC treatment on women with fewer financial resources. Previous research also suggests lower rates of employment benefits such as paid sick leave among lower-wage workers and variations by individual demographics, as well as industry and occupation (39). Future studies should leverage qualitative methodologies to further investigate the relationship between income status and employment outcomes among cancer patients. It is also critical that financial and employment outcomes are understood from the standpoint of social determinants of health and health equity. With this perspective, proposed solutions will mitigate the unintended consequence of exacerbating existing disparities in cancer care.

Both the American Society of Clinical Oncology and National Academy of Medicine have formally encouraged oncologists to discuss costs of care with patients before initiating treatment (40,41). The intent is to engender shared decision-making and a BC treatment decision process that is cost-sensitive and concordant with patients’ values, goals, and preferences (42). The costs of care were shown to be a major driver of surgical treatment decision-making among BC patients with a self-reported annual income less than $45 000 (2).

To support costs-of-care discussions with providers and to inform patients about the potential costs of treatment, recent federal initiatives have focused on improving price transparency, increasing access to online price estimators, and eliminating out-of-network surprise billing (43-45). Whether these initiatives will reduce CHE associated with cancer care remains unknown. Studies have only recently begun to assess other interventions, and financial navigators may be one promising solution (29,46-48). Financial navigation refers to the provision of individualized assistance to patients, families, and caregivers to overcome the financial barriers to timely, high-quality care (29). It also entails helping patients understand the financial aspects of their care, budget appropriately, and manage their employment and disability benefits (29).

Relying on patients to independently seek financial assistance is unlikely to be effective due to an identified lack of awareness, perceptions of ineligibility, and a fear of negative consequences (49). Therefore, future studies should seek to understand the supporting clinical workflows, barriers, and facilitators that engender the integration of financial navigation into oncology practice (50).

Our study also provides additional evidence that the ACA improved the insurance status among BC patients, a finding consistent with other major studies (18,51). Despite reduced uninsurance through the study period, we also showed an increased rate of CHE despite ACA implementation. This suggests that ongoing changes in cost-sharing arrangements (ie, HDHPs) have intensified the financial burden of cancer services for patients (33,52). It remains unlikely that clinical algorithms for BC patients will evolve to be less complex or expensive in the future. Therefore, it is worth considering the merits of value-based insurance design (ie, covering high-value cancer services on a pre-deductible basis) in BC care (53).

A more comprehensive evaluation of potential policy solutions must include both quantitative and qualitative elements, as well as comprehensive understanding of the barriers and facilitators that exist for the policy to have its intended impact. For example, if a policy is implemented at the state level (eg, Medicaid expansion), then an ex ante impact assessment should leverage state-level data and rigorous statistical methods to eliminate confounding factors and secular trends unrelated to the policy. Also, national and state-level data do not capture patient-level details about the impact of policies. For instance, insurance expansion may improve access, but issues related to health literacy and distrust in the medical establishment or government may affect uptake. One consideration would be using probability-based sampling to capture the lived experiences and perspectives of individuals through either surveys or qualitative interviews in the subsequent months and years after a policy.

An important limitation of this study is that the patients were not directly asked specifically whether or not their financial, insurance, or employment outcomes were related to their diagnosis. To account for this limitation, we included a matched control sample of similar women without BC. However, it remains possible that groups are different in unmeasured ways that affect the comparisons and point estimates for our primary outcomes. MEPS does not capture caregiver, transportation, and parking costs that may have an important impact on patients and families. MEPS does not provide any way to understand the stage of cancer and its relation to the outcomes of interest. This is an important area of future investigation. Since comorbidities were not a central focus of this study and ICD codes were not reported in full detail in MEPS, we did not attempt to impute comorbidities from ICD codes with the MEPS data and then estimate a comorbidity index for our analyses. Therefore, a comprehensive understanding of how comorbidities are associated with the outcomes of interest is limited. Finally, the sample is limited to individuals with expenditures related to a BC diagnosis within a given year, and we were unable to determine the time from initial BC diagnosis and survey date. Therefore, this study may not be representative of a newly diagnosed sample and may include individuals who have had breast cancer for several years and are still undergoing treatment and follow-up care, as well as individuals with previously treated cancer who are undergoing surveillance.

Although our understanding of BC pathophysiology and treatment have improved drastically over the past few decades, the ensuing benefits are not shared by patients equitably, and the financial and employment impact of therapies may be underappreciated. Our study underscores the challenges ahead in improving the delivery of high-quality, affordable, and equitable cancer care. A multilevel understanding of these issues is needed to inform strategies to improve quality of life and survivorship for these patients, as well as to ensure comprehensive evaluation of potential policy solutions.

## Data Availability

All data are publicly available for download by the Agency for Healthcare Research and Quality (AHRQ) through the following website: https://meps.ahrq.gov/mepsweb/.
